# Distinguishing COVID-19 from seasonal influenza in patients under age 65 years—a retrospective observational cohort study comparing the 2009 influenza A (H1N1) and 2022 SARS-CoV-2 pandemics

**DOI:** 10.3389/fcimb.2023.1179552

**Published:** 2023-07-18

**Authors:** Wen Zhong, Yisong Wu, Wenxiang Yue, Jiabin Fang, Baosong Xie, Nengluan Xu, Ming Lin, Xiongpeng Zhu, Zhijun Su, Yusheng Chen, Hong Li, Hongru Li

**Affiliations:** ^1^ Department of Respiratory and Critical Care Medicine, Fujian Shengli Medical College, Fujian Medical University, Fujian Provincial Hospital, Fuzhou, China; ^2^ Department of Hematology, Quanzhou First Hospital, Quanzhou, China; ^3^ Department of Infectious Diseases, Quanzhou First Hospital, Fuzhou, China; ^4^ The School of Nursing, Fujian Medical University, Fuzhou, China; ^5^ Shengli Clinical Medical College of Fujian Medical University, Fuzhou, China; ^6^ Fujian Provincial Key Laboratory of Medical Big Data Engineering, Fujian Provincial Hospital, Fuzhou, China

**Keywords:** SARS-CoV-2, COVID-19, H1N1, influenza A, non-older adult patients, clinical characteristics

## Abstract

**Introduction:**

This study explored the differences in clinical characteristics between the 2009 pandemic influenza A (H1N1) and SARS-CoV-2 BA.2 variant (Omicron) infections in patients younger than age 65 years, to improve identification of these diseases and better respond to the current epidemic.

**Methods:**

Data from 127 patients with the 2009 pandemic influenza A (H1N1) diagnosed between May and July of 2009 and 3,265 patients with Omicron diagnosed between March and May of 2022 were collected. Using a 1:2 match based on age (difference <2 years), sex, and underlying diseases, data from 115 patients with the 2009 pandemic influenza A (H1N1) infection (H1N1 group) and 230 patients with SARS-CoV-2 Omicron BA.2 infection (Omicron group) were analyzed. The clinical manifestations were compared between the groups, logistic regression was performed to identify possible independent risk factors for each group, and multiple linear regression was used to analyze the factors predicting time for nucleic acid negativization (NAN).

**Results:**

The median [interquartile range] age of the two groups was 21 [11, 26] years. Compared with the H1N1 group, the Omicron group had: lower white blood cell counts and C-reactive protein levels; less fever, nasal congestion, sore throat, cough, sputum, and headache; and more olfactory loss, muscle soreness, and lactate dehydrogenase (LDH) abnormalities. Patients in the Omicron group used fewer antibiotics and antiviral drugs, and the time for NAN was longer (17 [14,20] VS 4 [3,5] days, P<0.001). Logistic regression showed that fever, cough, headache, and increased white blood cell count were more strongly correlated with the H1N1 group, while muscle soreness and LDH abnormalities were more strongly correlated with the Omicron group. Fever (B 1.529, 95% confidence interval [0.149,2.909], P=0.030) significantly predicted a longer time for NAN in patients with Omicron.

**Discussion:**

There are significant differences in clinical characteristics between SARS-CoV-2 Omicron infection and the 2009 pandemic influenza A (H1N1) infection. Recognition of these differences has important implications for clinical practice.

## 1 Introduction

The outbreak of the novel SARS-CoV-2 in 2019 (COVID-19) led to a global pandemic that infected 664 million patients and killed 6.7 million as of January 22, 2023 (World Health Organization, 2023). Because of its strong mutation capacity (Morales et al., 2021), although nations took measures to control its spread, their effects were limited. The Alpha variant emerged first, followed by the Beta and Delta variants. At present, Omicron variant, which was discovered in November 2021, is dominant worldwide (Yeo et al., 2023). Although morbidity and mortality from Omicron have been significantly lower compared with the previous variants (Wolter et al., 2022), it spreads rapidly (with an R0 value close to 10). Its high reinfection rate (Lundberg et al., 2022) and strong resistance to current vaccines (Zeng et al., 2022) pose severe challenges to epidemic prevention and control.

The influenza virus has long been the most common viral respiratory pathogen (Zhou et al., 2019). There have been several influenza pandemics in the last century (Harrington et al., 2021), including the influenza A (H1N1) pandemic that began in the spring of 2009 and caused about 284,400 deaths worldwide (Dawood et al., 2012). This level of casualties from the 2009 pandemic influenza A (H1N1) was a wake-up call, and its many similarities to COVID-19 (e.g., similar transmission routes, clinical manifestations, and transmission ranges) have served as a reference for managing the current pandemic.

Most previous studies have focused on the difference between wild-type COVID-19 strains and seasonal influenza (Heo et al., 2022; Yang et al., 2022b). However, COVID-19 is evolving, and the differences between Omicron and seasonal flu have been under-described. With the seasonal change (i.e., arrival of winter and spring), both Omicron and influenza A (H1N1) may occur at any time. In the current context, with most countries having canceled routine nucleic acid testing, it is of great significance for clinicians to be able to differentiate between these viral infections. Furthermore, most previous studies have focused on older adult patients (Bao et al., 2022; Lu et al., 2022), while the differences between Omicron and the 2009 pandemic influenza A (H1N1) are rarely reported in younger patients. Although infections in younger patients are often mild (Silva et al., 2022), they should nevertheless be identified. Therefore, the study goal was to identify the differences between patients under age 65 years who were infected with the influenza A (H1N1) during the 2009 outbreak and those with Omicron BA.2 in 2022.

## 2 Methods

### 2.1 Ethics statement

The study was reviewed and approved by the Ethics Committee of Fujian Provincial Hospital (ethics number: K2019-12-032) and the Ethics Committee of Quanzhou First Hospital (ethics number: No202212). Written informed consent to participate in this study was provided by the participant, their legal guardian, or their next of kin, including for the publication of any potentially identifiable images or data included in this article.

### 2.2 Study participants

From May to July of 2009, 127 patients were diagnosed with the 2009 pandemic influenza A (H1N1) in Fujian Province. Among these, 126 were confirmed positive by real-time reverse transcription polymerase chain reaction (RT-PCR) test of pharyngeal (i.e., nasal) specimens by the Fujian Provincial Center for Disease Control and Prevention (CDC) and one patient was diagnosed by >1:4 tracking serum antibody titer (assay reagent was Shanghai ZJ Bio-Tech Co., Ltd).

We also collected data on 3,265 patients with Omicron, who were diagnosed between March and May, 2022. Second-generation whole genome sequencing was performed by the Fujian Provincial CDC for positive SARS-CoV-2 specimens by fluorescence real-time RT-PCR (assay reagent manufacturer Daan Gene Co., Ltd.), confirming that the COVID-19 strain in Quanzhou from March to May, 2022, was Omicron BA.2.

Using age (difference <2 years), sex, and underlying diseases, we performed a 1:2 match between patients infected with the 2009 pandemic influenza A (H1N1) (H1N1 group, n=115) and those infected with SARS-CoV-2 Omicron (Omicron group, n=230).

### 2.3 Data collection

Clinical data were collected from electronic medical records using standardized data collection tables. Serological results included whole blood cells, biochemical tests, and C-reactive protein (CRP) tests. All results were measured within 24 hours after admission. The time for nucleic acid negativization (NAN) was defined as the days from the patient’s symptom(s) onset or first positive nucleic acid test result to their post-treatment negative nucleic acid test.

### 2.4 Clinical management

Omicron group patients who were asymptomatic or mild were quarantined in a mobile field hospital, and those with moderate or more severe symptoms were hospitalized for treatment. All patients with the 2009 pandemic influenza A (H1N1) were treated in-hospital. Both patient groups were treated according to the standard of care guidelines (Ministry of Health of the People’s Republic of China, 2009; General Office of National Health Commission of the People’s Republic of China, 2022). Nirmatrelvir-Ritonavir antiviral therapy was used for patients who met the indications. Antibiotic therapy was used for patients with bacterial infections.

### 2.5 Statistical analysis

Descriptive data are presented as median [interquartile range (IQR)] for continuous parameters and as frequency (percentage) for categorical variables. Wilcoxon rank sum testing was employed for numerical variables and Fisher’s exact probability test for categorical variables. Logistic regression was performed to identify independent risk factors for each group, and multiple linear regression was used to identify the factors influencing time for NAN in each group. Statistical analyses were performed using SPSS version 25.0 data analysis software. A two-tailed P value <0.05 was considered statistically significant.

## 3 Results

### 3.1 Baseline information

Baseline data for the two groups are shown in **Table 1**. Due to matching, the groups did not differ on these measures. The median age in both groups was 21 [11, 26] years; 36.52% of participants were <18 years, 63.48% were 18–65 years, and no patient was over age 65 years. Overall, 56.52% of the sample were men and 43.48% were women. Nor did the groups differ in rates of pregnancy, hypertension, diabetes, cardiovascular disease, chronic liver disease, respiratory disease, nervous system disease, metabolic system disease, chronic kidney disease, tumor, or other conditions. Within the Omicron group, the proportions of patients who received 0, 1, 2, or 3 vaccine doses were 16.52%, 6.52%, 50.87%, and 26.09%, respectively.

**Table 1:** Baseline characteristics of H1N1 and Omicron groups.

	H1N1 group		Omicron group	
	N1	Frequency/IQR	N2	Frequency/IQR
Total (n)	115	100.00%	230	100.00%
Age				
Median age	21 [11,26]		21 [11,26]	
<18	42	36.52%	87	37.83%
18–65	73	63.48%	143	62.17%
>65	0	0.00%	0	0.00%
Sex				
Male	65	56.52%	130	56.52%
Female	50	43.48%	100	43.48%
Underlying diseases				
Pregnancy	0	0.00%	0	0.00%
Hypertension	0	0.00%	0	0.00%
Diabetes	0	0.00%	0	0.00%
Cardiovascular diseases	0	0.00%	0	0.00%
Chronic liver diseases	0	0.00%	0	0.00%
Respiratory diseases	0	0.00%	0	0.00%
Neurological diseases	0	0.00%	0	0.00%
Hematological diseases	0	0.00%	0	0.00%
Chronic kidney diseases	0	0.00%	0	0.00%
Metabolic diseases	0	0.00%	0	0.00%
Tumor	0	0.00%	0	0.00%
Vaccine status (%)				
No vaccination	–	–	38	16.52%
One dose	–	–	15	6.52%
Two doses	–	–	117	50.87%
Three doses	–	–	60	26.09%

### 3.2 Comparison of clinical characteristics

Compared with the Omicron group, the H1N1 group had higher probabilities of fever (90.43% VS 34.35%; P<0.001), nasal congestion (12.17% VS 1.74%; P<0.001), sore throat (41.74% VS 21.30%; P<0.001), cough (68.70% VS 36.52%; P<0.001), expectoration (26.09% VS 14.35%; P=0.012), and headache (15.65% VS 0.87%; P<0.001), and lower probabilities of olfactory loss (0.00% VS 6.96%; P=0.002) and muscle soreness (4.35% VS 14.78%; P=0.004) (**Table 2**).

**Table 2:** Comparison of clinical characteristics between H1N1 and Omicron groups.

	H1N1 group		Omicron group		P-value
Total (n)	115	100.00%	230	100.00%	
Clinical features					
Fever n (%)	104	90.43%	79	34.35%	<0.001
Nasal congestion n (%)	14	12.17%	4	1.74%	<0.001
Sore throat n (%)	48	41.74%	49	21.30%	<0.001
Olfactory loss n (%)	0	0.00%	16	6.96%	0.002
Taste loss n (%)	0	0.00%	7	3.04%	0.100
Cough n (%)	79	68.70%	84	36.52%	<0.001
Expectoration n (%)	30	26.09%	33	14.35%	0.012
Fatigue n (%)	15	13.04%	36	15.65%	0.630
Dyspnea n (%)	0	0.00%	7	3.04%	0.100
Diarrhea n (%)	1	0.87%	9	3.91%	0.174
Vomiting n (%)	1	0.87%	0	0.00%	0.333
Headache n (%)	18	15.65%	2	0.87%	<0.001
Muscle soreness n (%)	5	4.35%	34	14.78%	0.004
Laboratory diagnostics					
WBC count (10^9^/L), Median [IQR]	5.49	[4.40,6.93]	4.85	[3.76,6.45]	0.008
Lymphocyte count (10^9^/L), Median [IQR]	1.51	[1.09,2.02]	1.58	[1.09,2.53]	0.161
Abnormal LDH, n (%)	21	18.26%	68	29.57%	0.023
CRP (mg/L), Median [IQR]	4.60	[2.30,10.45]	3.23	[0.51,6.84]	0.001
Imaging diagnostics					
Pneumonia n (%)	4	3.48%	16	6.96%	0.229
Treatment and prognostic indicator					
Antibiotic usage rate, n (%)	16	13.91%	2	0.87%	<0.001
Antiviral usage rate, n (%)	115	100.00%	2	0.87%	<0.001
Nucleic acid negative time (d), Median [IQR]	4	[3,5]	17	[14,20]	<0.001

For descriptive analyses, data are presented as median [interquartile range (IQR)] for continuous parameters and as frequency (percentage) for categorical variables. Wilcoxon rank sum testing was employed for continuous variables and Fisher’s exact probability test for categorical variables. Statistical analyses were performed using SPSS version 25.0 data analysis software.

**Figure 1:**
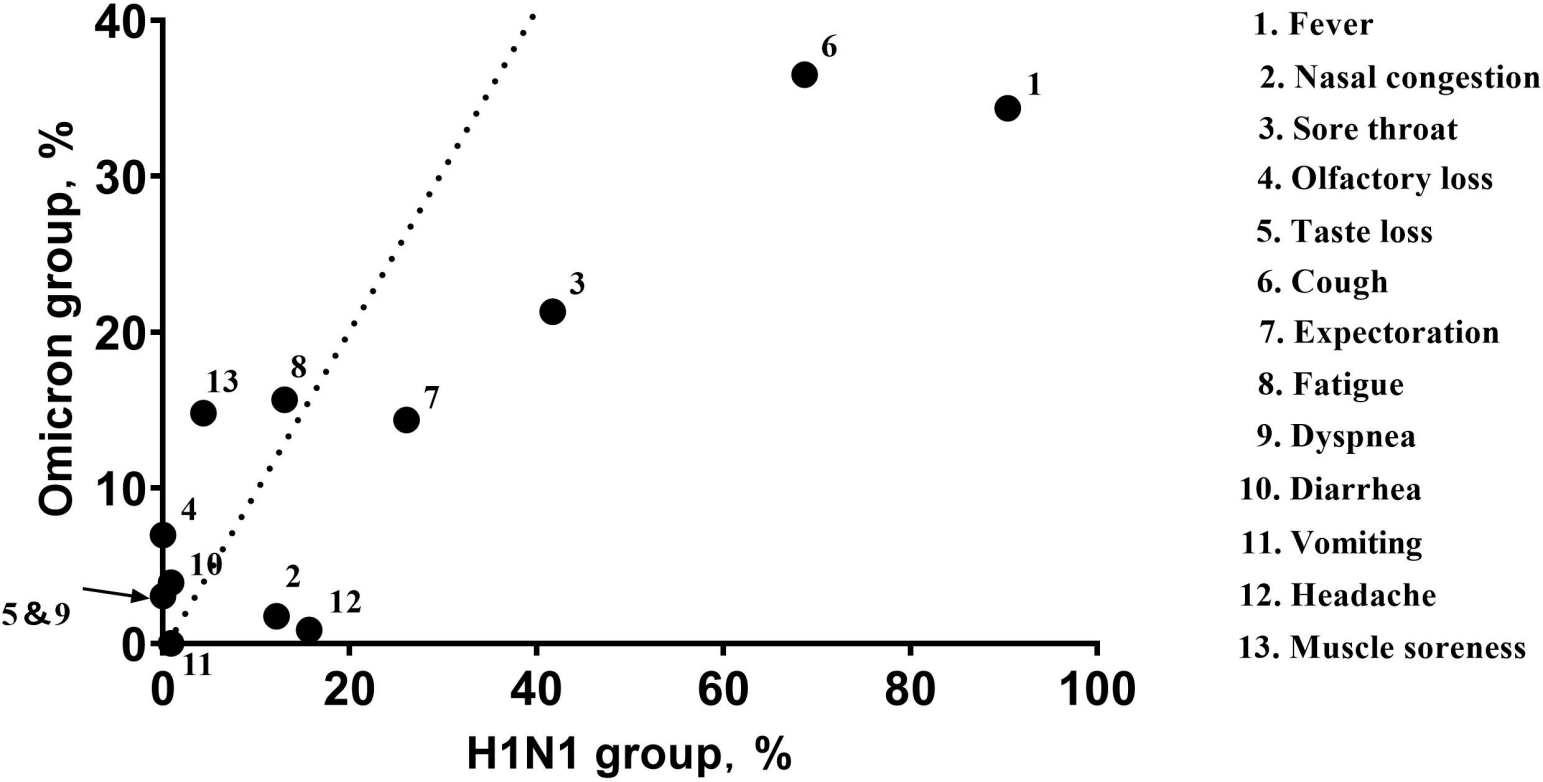
The abscissa of each point represents the frequency (percent) of the symptom within the H1N1 group, and the ordinate represents the frequency (percent) of the symptom in the Omicron group. The dotted line indicates equivalence of the abscissa and ordinate. The arrow indicates the completely coincident point. Fever (90.43% VS 34.35%; P<0.001), cough (68.70% VS 36.52%; P<0.001), and sore throat (41.74% VS 21.30%; P<0.001) were the most common symptoms in both groups; the Omicron group had lower symptom frequencies.

Within the H1N1 group, fever (90.43%), cough (68.70%), and sore throat (41.74%) were the most common symptoms. Similarly, the most common symptoms within the Omicron group were cough (36.52%), fever (34.35%), and sore throat (21.30%) (**Figure 1**).

**Figure 2:**
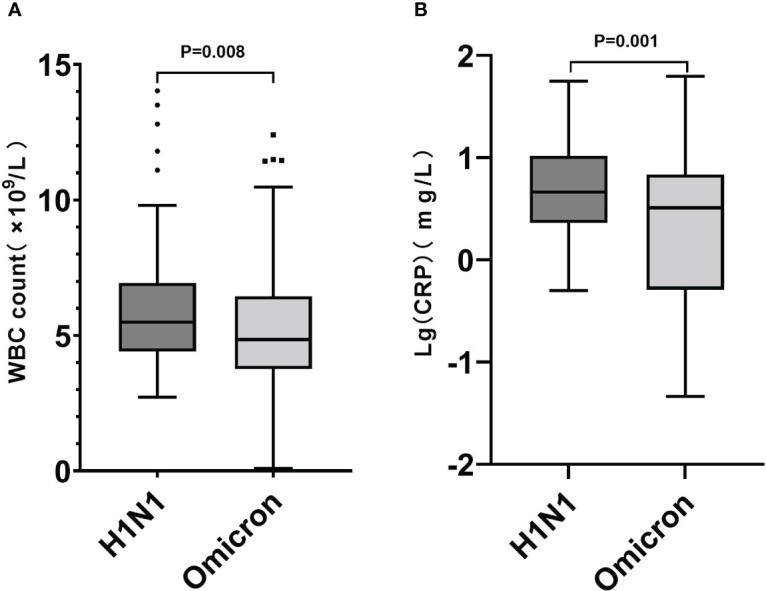
Box plot graphs revealing statistically significant differences in WBC counts **(A)** and CRP levels **(B)** between the Omicron and H1N1 groups. While most patients in both groups had normal WBC counts and CRP levels, the overall values in the H1N1 group were higher than those in the Omicron group (P<0.05).

The H1N1 group had higher white blood cell (WBC) count (5.49 [4.40,6.93] VS 4.85 [3.76,6.45]; P=0.008) and CRP levels (4.60 [2.30,10.45] VS 3.23 [0.51,6.84]; P<0.001) (**Figure 2**), while their lactate dehydrogenase (LDH) abnormal rate (18.26% VS 29.57%; P=0.023) was lower. There was not a significant between-group difference in lymphocyte count (1.51 [1.09,2.02] VS 1.58 [1.09,2.53]; P=0.161).

In terms of treatment and prognosis, the use of antibiotics (13.91% VS 0.87%; P<0.001) and antiviral drugs (100.00% VS 0.87%; P<0.001) was significantly higher in the H1N1 group than that in the Omicron group. The time for NAN (4 [3,5] VS 17 [14,20]; P<0.001) in the H1N1 group was significantly lower than in the Omicron group.

### 3.3 Risk factors

The statistically significant (P<0.05) clinical features, laboratory, and imaging diagnostics above were analyzed, using the Omicron group as the reference. The H1N1 group was more prone to fever (odds ratio [OR] 19.179, 95% confidence interval [CI] [8.820,41.708]; P<0.001), cough (OR 3.541, 95%CI [1.725,7.270]; P=0.001), headache (OR 15.695, 95%CI [2.288,107.679]; P=0.005), elevated WBC cell count (OR 1.190, 95%CI [1.027,1.378]; P=0.020), and less prone to muscle soreness (OR 0.051, 95%CI [0.013,0.200]; P=0.001) and LDH abnormalities (OR 0.393, 95%CI [0.194,0.795]; P=0.009) (**Table 3**).

**Table 3:** Comparison of risk factors between H1N1 and Omicron groups.

Factor	β	SE	Wald	P	OR	95%CI
Fever	2.954	0.396	55.538	<0.001	19.179	8.820–41.708
Nasal congestion	1.179	0.760	2.403	0.121	3.250	0.732–14.418
Sore throat	0.363	0.354	1.048	0.306	1.437	0.718–2.878
Cough	1.264	0.367	11.868	0.001	3.541	1.725–7.270
Expectoration	−0.193	0.459	0.176	0.675	0.825	0.335–2.030
Headache	2.753	0.983	7.852	0.005	15.695	2.288–107.679
Muscle soreness	−2.971	0.695	18.281	<0.001	0.051	0.013–0.200
WBC count	0.174	0.075	5.390	0.020	1.190	1.027–1.378
Abnormal LDH	−0.934	0.359	6.759	0.009	0.393	0.194–0.795
CRP	0.009	0.018	0.287	0.592	1.009	0.975–1.045

Multivariate logistic regression analysis was performed with Omicron group as the reference. Missing data were processed by multiple imputation.

### 3.4 Factors influencing time for NAN

For patients with the 2009 pandemic influenza A (H1N1), elevated WBC count significantly predicted a longer time for NAN (B 0.217, 95%CI [0.028,0.406]; P=0.025) and fatigue was associated with a shorter time for NAN (B −1.589, 95%CI [−2.646,−0.532]; P=0.004). For patients with Omicron, fever significantly predicted a longer time for NAN (B 1.529, 95%CI [0.149,2.909]; P=0.030) (**Table 4**).

**Table 4:** Factors influencing time for NAN.

	H1N1 group				Omicron group			
	Univariate		Multivariate		Univariate		Multivariate	
Variables	B	P value	B	P value	B	P value	B	P value
			(95%CI)				(95%CI)	
Fever	0.625	0.353			1.583	0.023	1.529 (0.149,2.909)	0.030
Nasal congestion	−0.133	0.826			0.816	0.749		
Sore throat	−0.009	0.982			0.066	0.935		
Olfactory loss	–	–			1.064	0.416		
Taste loss	–	–			1.233	0.525		
Cough	−0.038	0.929			0.514	0.458		
Expectoration	0.080	0.859			0.379	0.690		
Fatigue	−1.503	0.009	−1.589 (−2.646, −0.532)	0.004	1.346	0.141	1.077 (−0.724,2.878)	0.240
Dyspnea	–	–			0.054	0.978		
Diarrhea	0.531	0.803			0.054	0.975		
Vomiting	−2.496	0.241			–	–		
Headache	−1.089	0.044	−0.986 (−1.978, −0.006)	0.051	1.566	0.663		
Muscle soreness	0.968	0.318			1.166	0.214		
WBC count	0.325	0.001	0.217 (0.028,0.406)	0.025	−0.063	0.673		
Lymphocyte count	0.074	0.657			−0.093	0.665		
Abnormal LDH	0.639	0.170	0.182 (−0.666,1.029)	0.672	−0.015	0.983		
CRP	0.049	0.026	0.030 (−0.011,0.071)	0.152	0.040	0.297		
Pneumonia (Imaging)	2.099	0.050	1.473 (−0.525,3.470)	0.147	−0.347	0.791		
Antibiotic usage rate	1.809	0.001	1.076 (−0.100,2.252)	0.072	5.601	0.118	6.301 (−0.666,13.267)	0.076
Antiviral usage rate	–	–			−2.469	0.491		

The factors affecting time for NAN in the H1N1 and Omicron groups were analyzed by multiple linear regression. Statistically significant (P<0.200) differences on univariate analysis were included in the multivariate regression analysis. “–” indicates too many or too few values to perform single factor regression analysis. Missing data were processed by multiple imputation.

## 4 Discussion

The novel coronavirus, which began in 2019, continues to spread and mutate, hugely impacting the infectious disease load and burdening the global population (COVID-19 Excess Mortality Collaborators, 2022). During winter and spring, seasonal influenza virus infections are also common. This respiratory virus differs from SARS-CoV-2, but the clinical characteristics of patients it infects are similar, causing difficulties with diagnosis and treatment. There also exists a chance of coinfection (Yue et al., 2020). Therefore, the study goal was to learn from previous experience managing the 2009 influenza A (H1N1) pandemic (Chen et al., 2010) and compare it with the Omicron epidemic in Quanzhou (Li et al., 2022), to improve identification of these diseases.

Because of the younger age and fewer underlying diseases in the H1N1 group, the matched Omicron group also had such characteristics. The median age of the Omicron group was 21 [11,26] years, younger than the pre-matching population, whose median age was 36 [25,48] years (Li et al., 2022). The H1N1 group’s younger age may be related to their infection characteristics (Karageorgopoulos et al., 2011) (i.e., all 127 patients with influenza A [H1N1] were younger than 65 years). During the data collection period, the 2009 pandemic influenza A(H1N1) outbreak in China was still in the early stages and the influenza A(H1N1) vaccine had not yet been widely implemented. Consequently, none of this study’s 127 influenza A(H1N1) patients had received vaccination. On the contrary, a significant proportion of patients in the Omicron group had been vaccinated (16.52% received no doses, 6.52% received one dose, 50.87% received two doses, and 26.09% received three doses of the vaccine, respectively). Therefore, the immune status of the two groups could not be adequately matched. To elucidate the impact of the host immune system on viral infection, we conducted a review of relevant literature. Studies have demonstrated the efficacy of the 2009 pandemic influenza A(H1N1) vaccine in preventing disease (Tosh et al., 2010). However, research has shown that immune evasion after vaccination with COVID-19 (including against the Omicron subvariant) is common (Takashita et al., 2022a; Takashita et al., 2022b; Wang et al., 2022; Imai et al., 2023; Wang et al., 2023). For the Omicron variant and its sublineages (BA.1, BA.2, BA.2.12.1, BA.4, and BA.5), COVID-19 vaccines continue to be effective in preventing severe disease, but their effectiveness in preventing symptomatic infection is attenuated. An observational study conducted in the United States revealed that vaccine effectiveness against hospitalization was 79% within five months of receiving the last of three mRNA COVID-19 vaccine doses during the BA.1/BA.2 periods but decreased to 41% just five months after vaccination (Surie et al., 2022). Another study demonstrated that most individuals who were infection-naïve and only received the primary vaccine series had no detectable neutralizing activity against Omicron (Wf et al., 2022). Therefore, immune evasion may explain why many vaccine recipients still experience infection and exhibit typical clinical manifestations. Furthermore, this study specifically examined clinical manifestations in people under 65 years of age, with a very low incidence of severe disease caused by Omicron. Thus, the objective impact of vaccination on clinical manifestations was minimal. Additionally, it should be noted that in China, the number of people vaccinated against the 2009 pandemic influenza A(H1N1) is typically low under normal circumstances, while the administration of the Omicron vaccine is currently widespread. This discrepancy represents an objective reality. Although the lack of a matched vaccination status between the two groups in our study makes it challenging to eliminate the influence of immune status on viral infection, it better reflects the real-world differences in clinical manifestations between the 2009 pandemic influenza A(H1N1) and Omicron under current circumstances.

Herein, fever (90.43% VS 34.35%; P<0.001), cough (68.70% VS 36.52%; P<0.001), and sore throat (41.74% VS 21.30%; P<0.001) were the most common symptoms in both groups, consistent with previous studies (Novel Swine-Origin Influenza A (H1N1) Virus Investigation Team, 2009; Tiecco et al., 2022) showing respiratory symptoms to be primary. That the frequency of symptoms in the H1N1 group was higher than in the Omicron group is also consistent with a previous study that the virulence of Omicron was lower and more often caused asymptomatic infection (Yang et al., 2022a). The probability of muscle soreness (14.78% VS 4.35%) was higher with Omicron infection, suggesting that this variant was more likely to invade muscle tissue. This may be related to ACE-2 receptor expression in skeletal muscle cells and other cells in muscle (e.g., satellite cells, white blood cells, fibroblasts and endothelial cells). In addition to immune-mediated muscle damage, Omicron may directly invade and damage muscles (Paliwal et al., 2020).

The Omicron group’s olfactory and taste losses were major features differentiating them from the H1N1 group. Possible mechanisms for this may be that Omicron adheres to the motor cilia with help from the ACE-2 receptor, that it breaks through the periciliary layer (Wu et al., 2023), and that it infiltrates the olfactory epithelial tissue and induces local an inflammatory response (Khurana and Singh, 2022), eventually causing microvascular and axonal changes (Ho et al., 2022) that affect olfactory-related gene expressions (Zazhytska et al., 2022). Studies have also shown that nasal tissue responses to SARS-CoV-2 infection are more extensive than to the influenza virus, including maturation and activation of immune cells in both innate immunity and adaptive immunity (Alfi et al., 2021), which may lead to a stronger immune response in the nasal mucosa and cause greater damage, reducing olfactory function. Omicron can also enter specific epithelial taste cells through the ACE-2 receptor, destroying normal taste function, (Doyle et al., 2021) or affect the oral symbiotic flora to change the immune status and induce cytokine storms, ultimately damaging the taste nerve and destroying its function (Xu et al., 2022). Although there is no effective treatment for this sensory loss (Khurana and Singh, 2022), most patients (>95%) recover completely or nearly completely within six months after the acute phase (Tan et al., 2022).

Herein, the H1N1 group had higher WBC counts and CRP levels, possibly suggesting that the inflammatory response caused by the 2009 pandemic influenza A (H1N1) is more severe. Distinct from previous studies (Yang et al., 2022b), WBC counts, lymphocyte counts and CRP levels in the Omicron group were within normal ranges, possibly related to this sample’s younger age (i.e., they may have relatively stronger immunity compared with older adult patient populations). The diversity of T cells, which play an important role in viral elimination (Kumar et al., 2018), gradually decreases and shows a sharp decline after age 40 years (Simnica et al., 2019). One study showed that in patients with Omicron, younger people had higher levels of neutralizing antibodies (Liang et al., 2022). These differences may allow faster and more efficient viral clearing in younger patients, without significantly affecting the immune system, so that their lymphocyte counts remain within the normal range; this is supported by evidence that clinical symptoms among younger patients are relatively mild compared with those of older adults (Davies et al., 2020). Herein, the Omicron group had a higher probability of LDH abnormality (29.57% VS 18.26%), possibly related to the wide tissue distribution of the ACE-2 receptor (Li and Qin, 2021). Omicron may cause tissue damage through this receptor, leading to muscle soreness and abnormal LDH, though the specific mechanism remains unclear.

Herein, among patients under age 65, those with the 2009 pandemic influenza A (H1N1) or Omicron both had a relatively low incidence of pneumonia (3.48% and 6.96%, respectively), and their symptoms were mainly of the upper respiratory tract. The low incidence of pneumonia may be related to the sample’s age, as younger patients have more mild infections (Davies et al., 2020), or to the relatively strong nasal and weakened pulmonary tropism of Omicron (Wu et al., 2023). In addition, since H1N1 is an influenza virus, a specific antiviral (oseltamivir) was available when it broke out. High utilization of antiviral drugs (100.00% VS 0.87%; P<0.001) may have led to a lower incidence of pneumonia. Furthermore, the use of antibiotics in the H1N1 group was higher than in the Omicron group, indicating a higher probability of bacterial coinfection, consistent with a previous study (Centers for Disease Control and Prevention (CDC), 2009).

Logistic regression analysis showed that fever, cough, headache, and 1-unit (10^9^/L) increased WBC count probabilities in patients with the 2009 pandemic influenza A (H1N1) were 18.689, 3.853, 16.649, and 1.228 times higher, respectively, than in those with Omicron. This differs from previous studies (Li et al., 2021; Lv et al., 2021) in which more symptoms like fever and cough occurred with the original COVID-19 strain infection. However, the 2009 pandemic influenza A (H1N1) infection was less likely to cause muscle soreness and LDH abnormalities (probabilities of 0.102 and 0.373 times that of Omicron, respectively). These may be related to damage from Omicron to many tissues, including muscle, from widely distributed ACE-2 receptors (Paliwal et al., 2020; Li and Qin, 2021).

Herein, the time for NAN in the Omicron group was significantly higher compared with the H1N1 group (17 [14,20] VS 4 [3,5] days, P<0.001). NAN means that the patient is no longer an infection source; thus, this sample’s longer time for NAN shows that Omicron causes a longer infectious time and spreads faster compared with the 2009 pandemic influenza A (H1N1) (R0 values: 10 VS 2.75) (Brooks et al., 2010; Burki, 2022). This may be related to specific antibody productions and antiviral drug use. Due to long-term seasonal influenza epidemics, some people have cross-antibodies against the 2009 pandemic influenza A (H1N1) (Hancock et al., 2009). Moreover, the 2009 pandemic influenza A (H1N1)-specific antiviral drugs can quickly inhibit the virus, so that it is cleared more quickly. In contrast to Omicron, which often shows immune escape due to its strong mutation ability (Cao et al., 2022), and significantly reduced production of neutralizing antibodies in response to the Omicron strain (Liang et al., 2022). Regarding the use of antiviral medications, Roche’s oseltamivir data published in 2014 demonstrated that oseltamivir shortened the time to alleviation of symptoms by 17 hours in adults and 29 hours in children (Jefferson et al., 2014). We also examined literature regarding the time for NAN after Omicron infection. A study conducted in 2021 found that the remdesivir, hydroxychloroquine, lopinavir and interferon regimens had little to no effect in hospitalized patients with COVID-19, as indicated by overall mortality, initiation of ventilation and duration of hospital stay (WHO Solidarity Trial Consortium et al., 2021). Furthermore, a recent meta-analysis of Paxlovid (Amani and Amani, 2023), a specific drug for COVID-19, revealed that the Paxlovid group and the non-Paxlovid group exhibited significant differences in terms of mortality, hospitalization rate, and negative conversion time of PCR (mean difference [MD] = -2.46; 95% CI: -4.31 to -0.61). However, Paxlovid only reduced the PCR negative conversion time by 2.46 days. In summary, these pieces of evidence support the idea that although antiviral drugs can impact the time for NAN in different groups, the shorter time caused by the 2009 pandemic influenza A(H1N1) (as demonstrated in this study: 4 [3, 5] VS 17 [14, 20] days, P < 0.001) is primarily attributed to virus characteristics. Besides, the factors significantly predicting longer time for NAN herein included antibiotic use and increased WBC counts, possibly due to bacterial coinfection complicating these patients’ conditions (Krumbein et al., 2022). For patients with Omicron, fever is positively correlated with the time for NAN. This cumulative evidence suggests that if patients with influenza A (H1N1) have an elevated hemogram and patients with Omicron have a fever, clinicians should be prepared for a longer disease course, and that early use of antiviral drugs may help shorten these patients’ times for NAN (Zhou et al., 2010; Weng et al., 2023).

This study was not without limitations. First, since all patients were under age 65 years, the findings may not generalize to those with high risk from advanced age and underlying conditions. Second, as a real-world study, differences in clinical practices between the pandemic periods mean unavoidable confounding variables (e.g., host immune levels, vaccination status, antiviral use) and may reflect the clinical features of the diseases. Finally, significant biological differences between the 2009 pandemic influenza A (H1N1) and Omicron mean that factors influencing time for NAN are complex and likely bias the analyses.

## Data availability statement

The original contributions presented in the study are included in the article/supplementary materials, further inquiries can be directed to the corresponding author/s.

## Ethics statement

The studies involving human participants were reviewed and approved by the Ethics Committee of Fujian Provincial Hospital (ethics number: K2019-12-032) and the Ethics Committee of Quanzhou First Hospital (ethics number: No202212). Written informed consent to participate in this study was provided by the participants’ legal guardian/next of kin. Written informed consent was obtained from the individual(s) for the publication of any potentially identifiable images or data included in this article.

## Author contributions

HRL and HL were responsible for funding acquisition. HRL and WY were responsible for design conception. JF was responsible for formal analyses. WZ, YW, and JF performed data curation. HL and YC were responsible for acquiring resources. BX, NX, ML, and XZ verified the underlying data. YW and WZ prepared the original manuscript draft. All authors contributed to manuscript reviewing and editing. All authors contributed to the article and approved the submitted version.
